# Pediatric Myringoplasty using the Periosteum: An Institutional Overview

**DOI:** 10.1055/s-0043-1776001

**Published:** 2024-05-25

**Authors:** Al Hussein Awad, Mahmood A. Hamed

**Affiliations:** 1 Otorhinolaryngology Department, Faculty of Medicine, Sohag University, Sohag, Egypt

**Keywords:** periosteum, middle ear, myringoplasty, otitis media, outcomes

## Abstract

**Introduction** Myringoplasty is a common otologic procedure to restore the integrity of the tympanic membrane in cases of traumatic or pathologic perforations. Many grafting materials have been used with different techniques.

**Objective** In the present work, we evaluate the surgical and audiological outcomes of periosteal graft overlying the mastoid cortex through a retroauricular incision in a pediatric cohort.

**Methods** A retrospective study was carried out involving all children aged ≤ 16 years who underwent periosteal graft myringoplasty for the treatment of chronic suppurative otitis media with dry central perforation in our hospital from April 2019 to April 2021. All patients were followed up for one year to assess the anatomical success and functional outcomes by comparing the preoperative and postoperative (after six months) results of pure tone audiometry (PTA).

**Results** The sample was composed of 36 patients; 20 of them were female (55.6%) and 16 were male (44.4%) subjects, with ages ranging from 7 to 16 (mean: 12.7) years. Four patients underwent surgery in both ears (with an interval of 6 to 9 months). Out of 40 surgeries performed, 38 ears have shown anatomical success (95%). A highly significant improvement in hearing was obtained (the mean difference between the pre- and postoperative results of the PTA was of 14.6 ± 3.45 dB ( *p* < 0.001).

**Conclusion** We advocate the use of periosteal graft in the pediatric population as a good alternative for other types of grafts, with comparable and even better functional and anatomical outcomes.

## Introduction

The term *myringoplasty* was first described by Berthold in the end of nineteenth century; he succeeded in closing a tympanic membrane (TM) perforation using a full-thickness skin graft harvested from the forearm. Since then, many trials have been conducted using different biologic materials and autologous tissues, until the introduction of operative microscopy, which revolutionized otologic surgery. 1 2 Myringoplasty is defined as the standard surgical treatment for tubotympanic chronic suppurative otitis media (CSOM), which includes simple TM grafting without ossiculoplasty. 3

To restore the integrity of a perforated drum, numerous grafting materials have been used, including the temporalis fascia, cartilage, veins, fat, the perichondrium, and the periosteum. 4 5 6 7 The type of grafting material is a critical factor in the success of the surgical procedure. 4 The temporalis fascia ranks first among other grafting tissues, with success rates ranging from 93% to 97%. 6 Over the past few years, there has been a rising trend in the use of cartilage grafts as a good substitute for the temporalis fascia. Since it is stiffer, it can resist infection; however, this stiffness may have a negative impact on hearing gain. 5 6 In comparison to temporalis fascia and cartilage grafts, periosteum grafts have advantages over both, in that it is thicker than the fascia and thinner than cartilage, which enables their use in cases of otorrhea, Eustachian tube dysfunction, or revision surgery, with better hearing outcomes than those provided by thick cartilage. 8

In the pediatric population, myringoplasty requires special considerations, due to recurrent episodes of middle ear and upper respiratory tract infections. 9 To our knowledge, this topic has been rarely mentioned in previous research. In the present study, we aimed to evaluate our experience with the use of periosteal grafts in the repair of TM perforations in pediatric CSOM patients, discussing their demographics as well as their functional and anatomical outcomes.

## Methods

We performed a retrospective study involving children aged ≤ 16 years diagnosed with CSOM with dry central perforation (with no observation of aural discharge for 3 months or more), and periosteal myringoplasty was planned for them in our department from April 2019 to 2021. The study followed regional and institutional guidelines for human studies and was approved by the institutional Ethics Committee. A written informed consent was obtained from the patients' guardians. Detailed history was taken, and a full ear, nose, and throat (ENT) examination was performed, including endoscopic examination of the nose, nasopharynx and Eustachian tube, as well as a general examination and routine laboratory investigations. Patients with adenoid or adenotonsillar hypertrophy requiring surgery were excluded from our cohort. The ear examination included inspection, palpation, and otoscopic and microscopic examinations after dry cleansing or suction of wax and any visible aural discharge. The hearing evaluation was performed using tuning fork tests. Pure tone audiometry (PTA) for air and bone conduction (pure tone average air-bone gap [PTA-ABG]) in the frequencies of 0.5 kHz, 1 kHz, 2 kHz, and 4 kHz was performed for all patients within 1 week preoperatively and 6 months postoperatively. Patients in active, quiescent stages of CSOM, with aural polyps or cholesteatoma, were excluded from our cohort. In addition, patients with traumatic perforation, whether accidental, iatrogenic, or perforation following the insertion of tympanostomy tubes, ossicular discontinuity, tympanosclerosis, sensory neural hearing loss, and previous ear surgery were also excluded.

### Surgical Technique

**Fig. 1:**
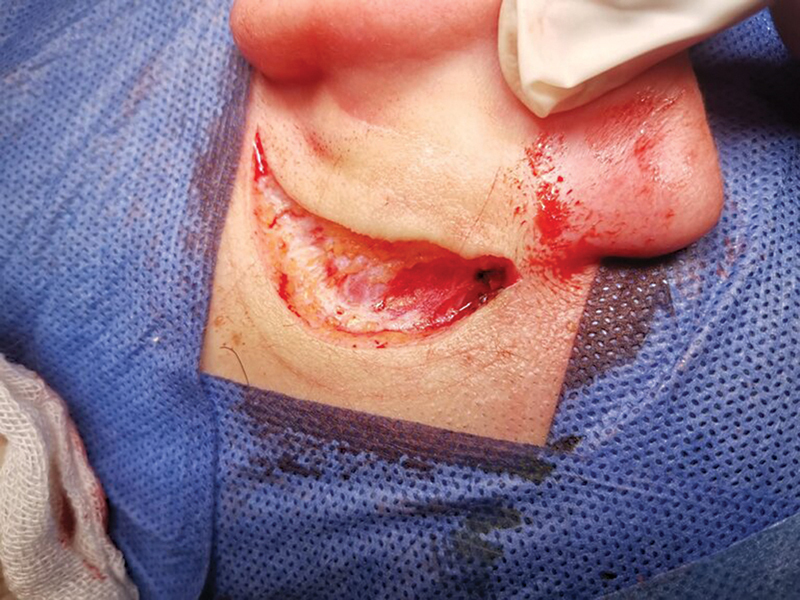
Postauricular incision.

**Fig. 2:**
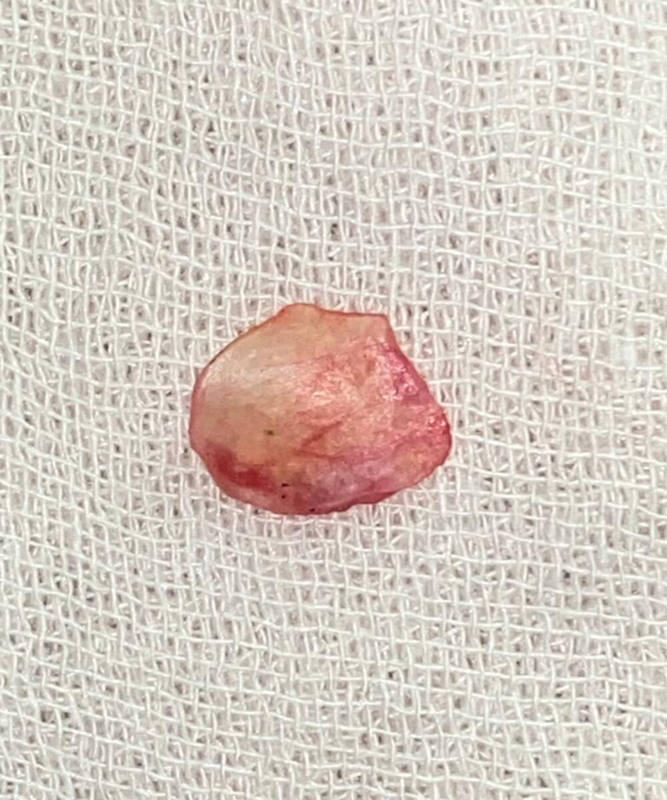
Periosteal graft.

Surgery was performed under anesthesia by senior ENT surgeons. A postauricular incision was performed and deepened until reaching the periosteal layer covering the mastoid cortex ( Fig. 1 ). Then, the graft was gently harvested with a scalpel and periosteal elevator, crushed in a graft forceps, and carefully fashioned according to the size of the perforation ( Fig. 2 ). The posterior meatal wall was elevated until reaching the tympanic annulus, and the middle ear was entered. Refreshing of the perforation edges was performed with a microscopic needle and round knife until a bleeding surface was obtained all around. Syringing of the Eustachian tube with saline through a wide pore cannula was performed to confirm its patency. Ossicular chain mobility was assessed.

After preparation, the graft was placed through the underlay technique (underneath the malleus handle and medially to the remnants of the tympanic membrane) under microscopic vision. In cases of amputated or short malleus handle, the graft was placed over the handle. Small pieces of absorbable gelatin sponge (Gelfoam, Pharmacia & Upjohn Company LLC, Kalamazoo, MI, United States) were placed layer by layer after the replacement of the posterior meatal wall. Then, a petrolatum (Vaseline, Unilever, London, United Kingdom) gauze impregnated with antibiotic ointment was placed in the external auditory canal (EAC) and the wound was closed. A tight sterile dressing was placed over the wound for 48 hours. Both intraoperative and postoperative complications (during follow-up visits) were reported if present.

### Follow-up

Patients were discharged the day after the surgery, and in the early postoperative follow-up, systemic antibiotics were prescribed for ten days, the dressing was removed after two days, and the petrolatum gauze was removed after ten days.

**Fig. 3:**
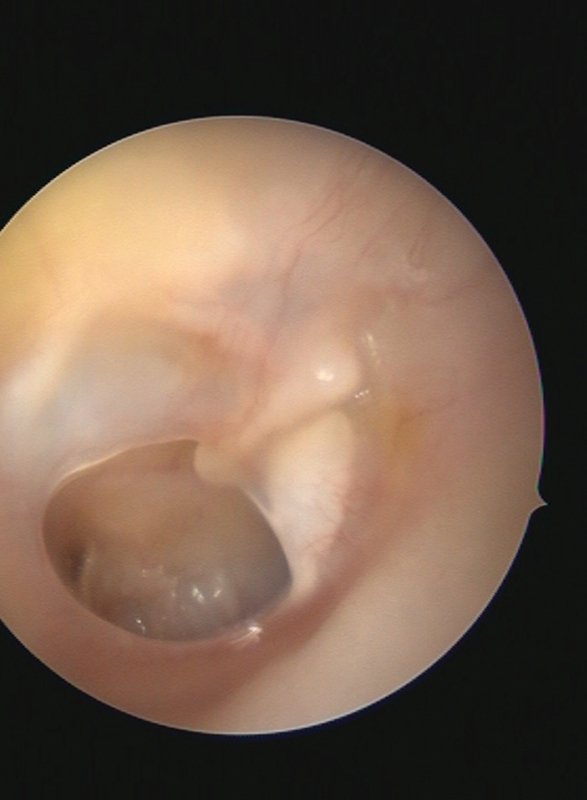
Preoperative left tympanic membrane with dry central perforation.

**Fig. 4:**
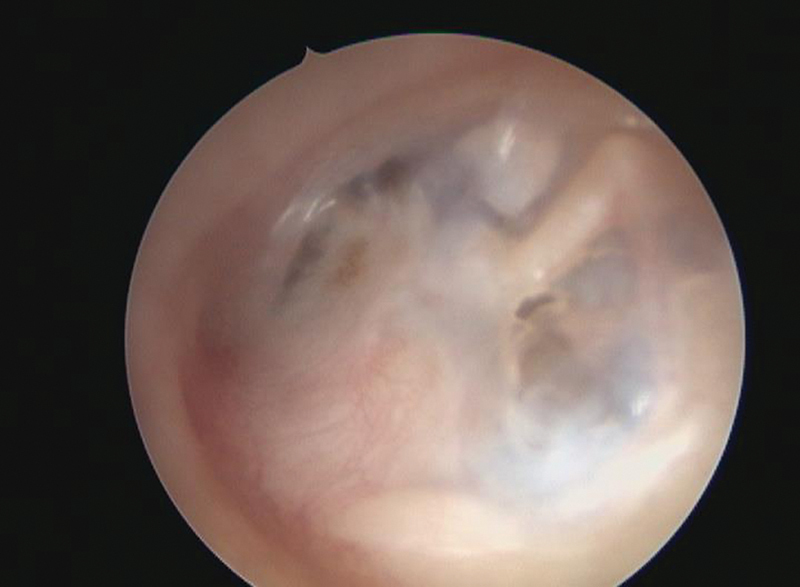
Postoperative (after six months) left tympanic membrane of the same patient.

**Fig. 5:**
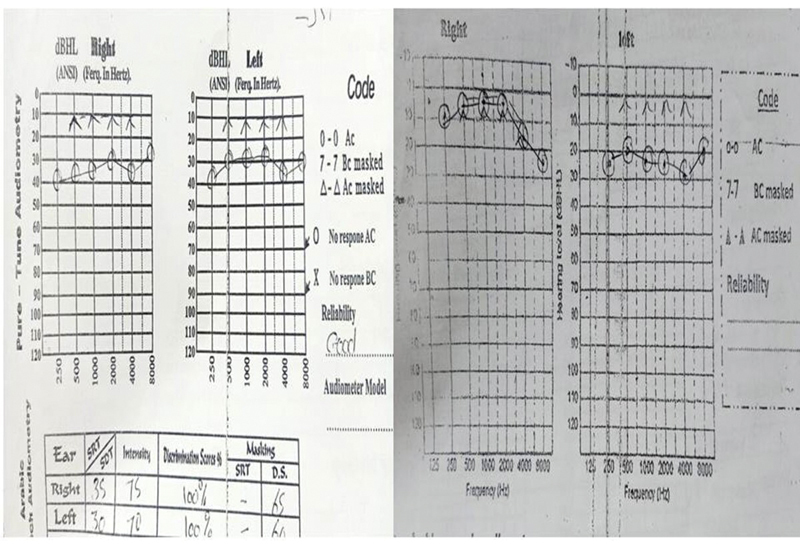
Pre- and postoperative (after six months) audiometry of a patient operated on in the right ear, showing complete closure of the air-bone gap.

All children were followed up for one year to evaluate graft healing; anatomical success was assessed monthly for the first six months; then, every two months for the next six months ( Figs. 3 , 4 ), and PTA was performed after six months to evaluate the hearing gain (functional success) ( Fig. 5 ).

### Statistical Analysis

Te categorial variables were expressed as numbers and percentages, whereas the continuous variables, as mean ± standard deviation (SD) values. The Student *t* -test was used to calculate the difference between the pre- and postoperative audiometric results. All analyses were performed using the IBM SPSS Statistics for Windows (IBM Corp., Armonk, NY, Unted States) software, version 19, and significance was set at *p* < 0.05.

## Results

The present study included 36 patients, 16 male (44.4%) and 20 female (55.6%) subjects with ages ranging from 7 to 16 (mean ± SD = 12.7 ± 4.5) years. The disease (CSOM) was unilateral in 21 patients (58.3%) and bilateral in 15 patients (41.7%). The main complaints were recurrent ear discharge (91.7%), hearing loss (86.1%), tinnitus (13.9%), and recurrent otalgia (16.7%). The procedure was performed in the right ear in 18 patients (45%), in the left ear in 22 cases (55%), and bilaterally (with an interval of 6 to 9 months) in 4 patients. Anatomical success and graft taking were assessed in 38 out of 40 ears (95%). Regarding the hearing gain, we found a highly significant difference between the preoperative (mean ± SD = 34.42 ± 4.51) and postoperative ABG (mean ± SD = 19.83 ± 4.68), with a mean improvement in hearing of about 14.6 ± 3.45dB ( *p* < 0.001) ( Table 1 ).

**Table 1:** Demographic data, anatomical and functional outcomes of the study sample

Number of patients (n)	36
Gender (n; male/female)	16/20
Age in years (mean ± SD)	12.7 ± 4.5
CSOM involved side (n; unilateral/bilateral)	21/15
Symptoms	
*Ear discharge (%)*	91.7%
*Hearing loss (%)*	86.1%
*Tinnitus (%)*	13.9%
*Otalgia (%)*	16.7%
Operated ear (n; right/left)	18/22
Graft uptake: n (%)	38 (95%)
Preoperative ABG in dBs (mean ± SD)	34.42 ± 4.51
Preoperative ABG in dBs (mean ± SD)	19.83 ± 4.68
Improvement in hearing in dBs (mean ± SD)	14.6 ± 3.45 ( *p* < 0.001)

Abbreviations: ABG, air-bone gap; CSOM, chronic suppurative otitis media; dBs, decibels, SD, standard, deviation.

## Discussion

Since it was first described by Berthold, various grafting tissues have been used for myringoplasty to obtain an intact TM after trauma or CSOM. The grafts vary in terms of their ease of harvesting, preparation time, ease of placement, viability, uptake, and hearing improvement. Of these autologous tissues, the temporalis fascia and cartilage are the commonest in the recent practice, with comparable uptake and hearing outcomes, which reach more than 90%. 6 8 10 The periosteum has been long used; however, still in a limited fashion. It presents many advantages compared to the temporalis fascia in TM repair, due to its consistency, elasticity, easier manipulation, and the fact that it matches the fibrous layer of the ear drum, which facilitates its uptake. In addition, the periosteum can resist well in the first few days after transplantation due to its very low metabolic requirements. Moreover, it has been proved to act as an excellent template for vascularization, 11 and it can be used in cases of discharging ears and revision. The periosteum is characterized by its availability (as it can be harvested in the same incision), sufficient quantity, excellent contour, and good tensile strength. 8

To the best of our knowledge, the mentions of the use of the periosteum in pediatric CSOM in previous research are scarce. Some authors have reported 7 8 12 their experience with periosteal myringoplasty, but not in patients in a specific age group. In addition, most of their study samples were composed of adults. 7 8 12 Proper selection of a suitable graft is crucial, especially in pediatrics, due to recurrent episodes of respiratory tract infection, which threaten surgical success. 9 Our results have shown an excellent graft uptake (95%), which are similar to those observed by ElTaher et al. 7 (93%), Rao et al. 8 (96%) Elmoursy and Elbahrawy 12 (95%), and ElBatawi et al. 13 (93%). These results are also comparable to those found for other types of grafts, whether temporalis fascia or cartilage grafts. 6 10 12 13

The functional success of periosteal myringoplasty was evaluated by comparing the mean preoperative (34.42 ± 4.51) and postoperative (19.83 ± 4.68) ABG with the mean improvement in hearing (14.6 ± 3.45dB). The results were in line with those found by ElTaher et al. 7 and ElBatawi et al., 13 who reported improvements in hearing of about 11dB 6 months postoperatively in patients who underwent periosteal myringoplasty. These functional outcomes are comparable and superior to those reported for other grafting materials. 6 12 13 Regarding the causes of surgical complications and failure in pediatric myringoplasty, several factors have been reported in the literature, 9 14 15 including age (younger age groups showed higher failure rates), timing of the surgery (operating on wet ears increases the failure rate), condition of the upper respiratory tract, contralateral ear, presence of tympanosclerosis, and size and site of the perforation. In the present series, there were two cases of graft failure, which was attributed to the occurrence of early postoperative infection and missed follow-up visits.

In our opinion, periosteal graft is a very good option in pediatric myringoplasty, as it presents advantages compared to other types of grafting materials. Finally, the limitations of the present study include its relatively small sample size and the lack of a control group. In addition, a longer follow-up period would have strengthened our results.

## Conclusion

We advocate the use of periosteal graft in the pediatric population as a good alternative for other types of grafts, with comparable and even better functional and anatomical outcomes.
